# Accuracy in Patient Understanding of Common Medical Phrases

**DOI:** 10.1001/jamanetworkopen.2022.42972

**Published:** 2022-11-30

**Authors:** Rachael Gotlieb, Corinne Praska, Marissa A. Hendrickson, Jordan Marmet, Victoria Charpentier, Emily Hause, Katherine A. Allen, Scott Lunos, Michael B. Pitt

**Affiliations:** 1 University of Minnesota Medical School, Minneapolis; 2 Department of Pediatrics, University of Minnesota, Minneapolis; 3 M Health Fairview Masonic Children’s Hospital, Minneapolis, Minnesota; 4 Biostatistical Design and Analysis Center, Clinical and Translational Science Institute, University of Minnesota, Minneapolis

## Abstract

**Importance:**

Despite acknowledging that medical jargon should be avoided, health care practitioners frequently use it when communicating with patients.

**Objective:**

To characterize the understanding of common medical jargon terms by surveying a cross section of the general public and studying phrases that have established meanings in regular usage but different meanings in a medical context (eg, negative and positive test results).

**Design, Setting, and Participants:**

In this cross-sectional study, participants indicated their understanding of phrases that may have different meanings in medicine than in colloquial English via a mix of short answer and multiple choice questions. Several questions included paired phrases to assess for differences in understanding with or without jargon. Volunteers were recruited at the 2021 Minnesota State Fair near St Paul, Minnesota. An electronic survey was given to a volunteer sample of 215 adults (>18 years) who did not work or train to work in the medical field and spoke and read English.

**Exposures:**

Completing a written or verbal survey.

**Main Outcomes and Measures:**

The main outcome was an accurate understanding of the medical terminology. Free-text responses were coded by 2 researchers for comprehension. Secondary outcomes looked for associations between volunteer demographics and understanding.

**Results:**

The 215 respondents (135 [63%] female; mean [SD] age, 42 [17] years) demonstrated a varied ability to interpret medical jargon phrases. For example, most participants (207 [96%]) knew that negative cancer screening results meant they did not have cancer, but fewer participants (143 [79%]) knew that the phrase “your tumor is progressing” was bad news, or that positive lymph nodes meant the cancer had spread (170 [67%]). While most (171 [80%]) recognized that an unremarkable chest radiography was good news, only 44 participants (21%) correctly understood that a clinician saying their radiography was impressive was generally bad news. In each of the paired phrases comparing jargon vs nonjargon approaches, the nonjargon phrase was understood significantly better (*P* < .001).

**Conclusions and Relevance:**

These findings suggest that several common phrases are misunderstood when used in a medical setting, with the interpreted meaning frequently the exact opposite of what is intended.

## Key Points

**Question:**

How well do adults understand common phrases clinicians use when communicating with patients?

**Findings:**

In this cross-sectional study that surveyed 215 adults, participants frequently misunderstood and often assigned meaning opposite to what the clinician intended.

**Meaning:**

These findings suggest that use of common medical phrases may lead to confusion among patients affecting health outcomes.

## Teaser

This cross-sectional study assesses patient understanding of common medical jargon terms using results of a survey conducted at the Minnesota State Fair.

## Introduction

Health care professionals regularly use jargon when communicating with patients, despite acknowledging that it should be avoided.^1,2,3,4,5,6,7^ Though this medical language may facilitate communication between health care professionals, its use with patients can introduce confusion that may have serious consequences.^8,9,10,11^ The mismatch between our intent to avoid jargon and the reality of our frequent use of it has been called jargon oblivion.^12^ One potential reason for this disconnect is that, as health care professionals, we simply assume our patients understand the terminology we are using. No matter how intentional we are about minimizing jargon, we will not avoid using words and phrases that we fail to recognize as jargon in the first place. Accordingly, by better understanding what medical terms and phrases patients do or do not comprehend, we can expand our jargon identification toolkit and ultimately improve our communication with patients.

Previous studies have shown that while technical terminology, abbreviations, and acronyms are the most commonly used forms of jargon, several other types of jargon are also used frequently.^1,2^ These include terms that have a well-understood meaning in common usage but often have a different meaning in medicine. For example, in most contexts, negative typically indicates something bad, such as negative feedback, negative viewpoints, or negative reviews. However, in the medical context, negative typically has a different or even opposite meaning, whereas a negative test result often signifies a favorable outcome.^12^ In this study, we aimed to characterize the understanding of common medical jargon terms and phrases by surveying a cross section of the public at the Minnesota State Fair. While others have demonstrated how the public rarely understands technical terminology and acronyms used in medicine,^6,7,8,11,13^ we aimed to assess understanding of the types of jargon that include words or phrases that have common meanings in regular usage and different meanings in medicine because these phrases may be particularly confusing to patients. We also wished to understand if certain demographic factors (ie, age, gender, or education) were associated with differences in understanding and if the method of administering the survey (ie, written vs verbal) changed how well respondents understood the phrases. Our hypothesis was that many of these terms will be poorly understood by adults in the general public regardless of age, gender, education, or survey method.

## Methods

We report our findings of this cross-sectional study using the Strengthening the Reporting of Observational Studies in Epidemiology (STROBE) reporting guideline. This study was approved by the University of Minnesota institutional review board (IRB). Participants were given a printed study information form that described the study objectives and risks and benefits of participating. Given that obtaining a signature would have been the only identifying information in the study, the IRB determined it was a lower risk to have verbal consent alone.

### Setting and Survey Design

We surveyed a cross section of the public during 3 days at the 2021 Minnesota State Fair. We developed a 13-question survey with a mix of open-ended and multiple-choice questions assessing jargon understanding of common phrases used in medicine as well as brief demographics (age, gender, education). To look for changes over time, we included some questions that had been studied previously, such as^13^:

Imagine you are a patient who has just had surgery for cancer. After surgery your doctor gives you your test results and tells you the following: “Your nodes are positive.”

What do you believe your doctor is telling you?

A) You are clear of cancer
B) The cancer has spread
C) The cancer has not spread
D) Don’t know

Additionally, we included several new questions assessing understanding of phrases, which include words with meanings outside of medicine that differ from their meaning when used as a medical context, such as “Your chest X-ray was unremarkable,” “The findings on the X-ray were quite impressive,” or “Neuro exam is grossly intact.” Respondents were asked to indicate if they felt these statements indicated good news, bad news, or they were unsure.

Free-text response questions asked participants to write what they felt a doctor would mean by phrases, such as “occult infection” or “bugs in the urine,” because these also include words that have different meanings outside of medicine. We also assessed understanding of the word “febrile” because we hear this used often with patients but were uncertain of how broadly it is understood.

We also chose to assess understanding of alternative phrases for 2 terms that, in our experience, can be confusing to patients: “negative” and “NPO”—the latter of which has been previously shown to be poorly understood by patients.^14^ To compare the understanding of different ways of sharing this information, we developed paired items and presented them at separate points in the survey. For example, in a paired set of questions, participants were first asked to write what they felt it would mean if a doctor said, “You will need to be NPO at 8 am,” followed later by a question with the phrasing “You are to have nothing by mouth at 4 pm.” The full survey text is available in the eAppendix in the Supplement. After each question, respondents were asked how confident they were that they were correct (ie, not at all confident, somewhat confident, quite confident, or certain). Statistical analysis took place from September 2021 to December 2021.

### Survey Delivery Method

We developed both a written and a verbal version of the survey. For the written survey, all content was presented in written form on REDCap, accessed through an iPad.^15^ In the verbal versions, the question prompts were provided in written format on an iPad, but the phrases being studied were read aloud by a voice actor, accessed by clicking on an audio file icon and listening through headphones. Participants who received the verbal version could replay a sound file as often as they wished. Two phrases were written out in both versions of the survey because the question prompt asked what a patient would think if they read a certain phrase in their medical notes (eg, “neuro exam is grossly intact” and “I am concerned this patient has an occult infection”).

### Data Collection

Attendees entering or passing by the University of Minnesota’s Driven to Discover research building^16^ were invited to participate in the survey in exchange for a backpack with the University logo. Participants were eligible if they were at least 18 years old, had no history of medical or nursing training, and were comfortable participating in an English-language survey. The survey was anonymous and voluntary, and participants were able to stop at any point during the survey and still receive the incentive. After consenting to participate, volunteers were randomized by a throw of a die to either a written or verbal form of the survey.

### Statistical Analysis

Multiple choice responses were coded as correct or incorrect. Free-text responses were coded for accuracy by 2 independent researchers (R.G. and C.P), adding a third researcher if consensus was not reached.^17^ We used descriptive statistics to summarize the survey results, calculating means and standard deviations for continuous variables, and counts and percentages for categorical variables. Demographics and survey questions were compared between written and verbal groups using a *t* test for age, Fisher exact tests for categorical variables, and Wilcoxon rank sum test for confidence questions. McNemar test was used to compare correct responses for the paired questions with different options for sharing the same information. The association of a correct response with demographics (ie, age, gender, education, and group) and confidence was examined with multivariable logistic regression models. Adjusted odds ratio (aOR) and 95% CIs were reported from these models. *P* values <.05 were considered statistically significant. SAS V9.4 (SAS Institute) was used for the analysis.

## Results

In this cross-sectional study, 215 volunteers completed the survey (116 written, 99 verbal). Respondents had a mean (SD) age of 42 (17) years, 140 (65%) had a bachelor's degree or higher, and 135 (63%) were female. All respondents completed the entire survey. The demographics were statistically similar across the survey type (Table 1). Because there was no significant difference in correct understanding between those who were given the written and verbal surveys for all but 2 questions (Table 2), the responses are grouped together regardless of the method of survey administration for the remainder of the analysis.

**Table 1.** Summary of Demographics by Group^**a**^

Characteristic	No. (%)			*P* value^b^
	Total (N = 217)	Verbal (n = 101)	Written (n = 116)	
Age, mean (SD) [range]	42.4 (17.1) [18-88]	42.7 (16.5) [18-73]	42.3 (17.8) [18-88]	.86
Gender				.16
Female	135 (62.8)	58 (57.4)	77 (67.5)	
Male	80 (37.2)	43 (42.6)	37 (32.5)	
Education				.13
Some HS	3 (1.4)	0	3 (2.6)	
HS or GED	20 (9.2)	5 (5.0)	15 (12.9)	
Associates	18 (8.3)	9 (8.9)	9 (7.8)	
Some college	35 (16.1)	16 (15.8)	19 (16.4)	
Bachelor’s	70 (32.3)	39 (38.6)	31 (26.7)	
Grad or professional	70 (32.3)	32 (31.7)	38 (32.8)	
Other	1 (0.5)	0	1 (0.9)	

Abbreviations: GED, general educational development; HS, high school.

^a^ No statistically significant differences in demographics between the 2 groups.

^b^ Two group *t* test for age and Fisher exact test for categorical variables.

**Table 2.** Accurate Understanding of Jargon Phrases by Cross Section of the Public

Phrase	Answered correctly by survey type, No. (%)			*P* value (verbal vs written survey)^a^
	Overall (N = 215)	Verbal (n = 99)	Written (n = 116)	
Your blood tests showed me that you do not have an infection in your blood.	208 (97.7)	99 (100)	109 (94)	.02^a^
Your cancer screening test came back and the results are negative.	207 (96.3)	96 (97.0)	111 (95.7)	.73
Your blood culture was negative.	186 (86.5)	88 (88.9)	98 (84.5)	.42
Your chest x-ray was unremarkable.	171 (79.5)	80 (80.8)	91 (78.4)	.74
We are halfway through your chemotherapy treatment and your tumor is progressing.	170 (79.1)	78 (78.8)	92 (79.3)	>.99
You are to have nothing by mouth after 4 pm.	162 (75.3)	76 (76.8)	86 (74.1)	.75
Your nodes are positive.	143 (66.5)	64 (64.6)	79 (68.1)	.66
Patient’s neuro exam is grossly intact.^b^	89 (41.4)	NA	NA	NA
Your urine tests are back and there were bugs in your urine.	62 (28.8)	32 (32.3)	30 (25.9)	.37
The findings on the x-ray were quite impressive.	44 (20.5)	21 (21.2)	23 (19.8)	.87
You will need to be NPO at 8 am.	24 (11.2)	11 (11.1)	13 (11.2)	>.99
Have you been febrile?	20 (9.3)	9 (9.1)	11 (9.5)	>.99
I am concerned the patient has an occult infection.	4 (1.9)	4 (4.0)	0 (0)	.04^a^

Abbreviations: NA indicates not applicable; NPO, nothing by mouth.

^a^ *P* value <.05 considered statistically significant.

^b^ This question was written out in both surveys because the prompt was about reading this statement in a medical record.

There was mixed understanding of which phrases were meant to convey good news vs bad news (Table 2). For example, of the 215 respondents, most respondents (207 [96%]) knew that negative cancer screening results meant they did not have cancer. However, fewer respondents knew that “your tumor is progressing” was bad news (170 [79%]) or that positive nodes meant their cancer had spread (143 [67%]). Only 89 (41%) of respondents correctly interpreted “neuro exam is grossly intact” as good news. Additionally, while most respondents (171 [80%]) recognized that an unremarkable chest radiography was good news, only 44 respondents (21%) correctly understood that a clinician saying their radiography was impressive was generally bad news. Few respondents accurately understood the prompts that required a free-text response. Sixty-two respondents (29%) correctly interpreted “bugs in the urine” as intending to convey a urinary tract infection, 20 (9%) knew what febrile meant, and 4 (2%) of respondents understood the phrase occult infection.

Full findings from the paired items are presented in Table 3. Significantly more respondents correctly interpreted the phrase nothing by mouth compared with the use of the acronym NPO (162 respondents [75%] vs 24 respondents [11%], respectively; *P* < .001). When comparing the understanding of the same concept (blood infection) through nonjargon (“blood test shows no infection”) vs jargon (“your blood culture was negative”), significantly more respondents correctly interpreted the nonjargon phrase than the jargon phrase (208 [98%] vs 186[87%], respectively; *P* < .001).

**Table 3.** Comparative Understanding of Paired Phrases by Cross Section of the Public

Phrase	Answered correctly (n = 215), No. (%)	*P* value^a^
NPO vs nothing by mouth		<.001
You will need to be NPO at 8 am.	24 (11.2)	
You are to have nothing by mouth after 4 pm.	162 (75.3)	
Negative vs do not have		<.001
Your blood culture was negative.	186 (86.5)	
Your blood test showed me that you do not have an infection in your blood.	208 (96.7)	

Abbreviation: NPO indicates nothing by mouth.

^a^ *P* value <.05 considered statistically significant.

In multivariable logistic regression models, there were a few statistically significant associations between demographics and understanding (Table 4). Notably, increasing age was associated with increased understanding of nothing by mouth and negative blood cultures but decreased understanding of the term impressive in the context of radiography findings. Two questions showed an association with increased understanding if the respondent had a graduate degree: the phrases nothing by mouth and unremarkable, and the acronym NPO and the term febrile were better understood by women.

**Table 4.** Statistically Significant Demographic Associations With Correct Understanding of Jargon Phrases on Multivariable Logistic Regression^a^

Phrase	Demographic association with correct understanding	Adjusted odds ratio (95% CI)	*P* value^b^
Your blood culture was negative	Older age (each year) associated with increased understanding	1.03 (1.00-1.06)	.03
The findings on the x-ray were quite impressive	Younger age (each year) associated with increased understanding	0.96 (0.94-0.99)	.002
You are to have nothing by mouth after 4 pm	Older age (each year) associated with increased understanding	1.03 (1.01-1.06)	.002
	Graduate degree associated with increased understanding compared with associate’s degree or lower	3.33 (1.39-7.99)	.007
	Bachelor’s degree associated with increased understanding compared with associate’s degree or lower	2.23 (1.00-4.95)	.049
Your chest x-ray was unremarkable	Graduate degree associated with increased understanding compared with associate’s degree or lower	3.45 (1.35-8.87)	.01
You will need to be NPO at 8 am	Female gender associated with increased understanding	5.65 (1.59-20.13)	.008
Have you been febrile?	Female gender associated with increased understanding	5.90 (1.31-26.71)	.02

Abbreviation: NPO indicates nothing by mouth.

^a^ Associations between the 3 surveyed demographics (ie, age, gender, and education) and each of the 13 questions were assessed with multivariable logistic regression models yielding 39 comparisons. The 7 associations which reached statistical significance are depicted here.

^b^ *P* value <.05 considered statistically significant.

In general, the more confident the respondents were in their answers, the more likely they were to be correct. Additionally, respondents were more confident about both of the approaches to sharing blood culture results if they heard the clinician say them vs if they read the phrase. The confidence in the accuracy of verbal vs written questions for “blood culture was negative” was mean (SD) of 3.0 (0.9; median, 3.0) vs 2.7 (0.8; median, 3.0; *P* = .04). The confidence in accuracy of verbal vs written questions for “blood test showed me you do not have an infection” was mean (SD) 3.6 (0.7; median, 4.0) vs 3.3 (0.8; median, 3.0; *P* = .003).

## Discussion

In this cross-sectional study, we found that terms and phrases commonly used in clinical settings remain frequently misunderstood. Many studies of jargon comprehension take place in a medical setting, either as observational studies^1,2,3,6,9^ or surveys of patients in clinics.^8,18,19^ We aimed to better capture a less clinically biased sample by surveying a cross section of the public at the Minnesota State Fair to determine their understanding of commonly used medical jargon. To our knowledge, this is the largest study of patients’ understanding of jargon and the first to compare the understanding of jargon vs nonjargon phrases.

Our testing of several phrases that had been studied previously yielded several notable differences, in most cases, with a higher proportion of our study sample demonstrating understanding. For example, in 2001, Chapman et al^13^ found that among a sample of 105 adults in the UK, only 52% understood that the phrase “the tumor was progressing” signified bad news. They noted that progress is interpreted as a good thing in most settings. In our study, 79% of respondents correctly understood this phrase as bad news, with an absolute increase in understanding of 27%. Similarly, 43% of the respondents in the Chapman et al study^13^ correctly understood that having positive nodes meant their cancer had spread vs 67% correct among our sample, an absolute increase of 24%.

Some of these differences may be accounted to cultural differences between the UK-based sample in the Chapman study^13^ and our sample in the US. Additionally, the high number of college graduates in our sample (65% with a bachelor’s degrees or higher) compared with the general population of the United States (35%)^20^ may be a factor in that difference, although Chapman et al^13^ also reported that “a large proportion of the sample was well educated.” However, it should be noted that we found no statistically significant association with the level of education in the accuracy of interpreting the jargon in our study sample for all but 2 survey questions, so this is unlikely to account for the differences fully.

Some changes in understanding may be the result of the COVID-19 pandemic. For example, we hypothesize that the widely used designations of negative and positive in the context of viral testing during the pandemic have increased the public’s understanding of these terms in the medical context, accounting for the near-universal understanding of negative cancer screening being considered good news in our study. However, it is worth noting that when comparing the understanding of the phrase “your blood test shows no infection” and “your blood culture was negative,” significantly more respondents correctly interpreted the phrase that avoided the word negative altogether.

The use of terms that mean something different in common usage than in a medical context—or medicalized English^1,2,12^—was a frequent cause of confusion in our study. More people believed that the phrase “had an occult infection” had something to do with a curse than understood that this meant that they had a hidden infection. Fewer than half knew that their neuro examination being “grossly intact” was a good thing, possibly because the word “gross” more often means “unpleasant” than “in general” in common usage. These terms may not necessarily be recognized by clinicians as jargon because they do not land in the commonly understood category of technical, medical terminology. However, they have been shown to be used frequently in clinical settings.^1,2^

Most published results of jargon understanding by adults involve having the respondents read a prompt a doctor might say and indicate their understanding.^7,8,13,18,19,21^ Given that, in most cases, jargon is spoken aloud by clinicians during patient encounters, we opted to assess understanding of both written and spoken forms of communication to determine if survey methods affected understanding. To our knowledge, our study was the first to directly compare understanding of medical jargon in written vs audio form. Overall, we did not observe any significant difference in understanding between these 2 delivery methods, which may support the less time-intensive, more frequently used written survey approach to assessing jargon understanding. We hope that future studies on medical jargon will further explore this observation at a larger scale, which may help provide important insights for optimizing the study of patient communication, particularly as patients increasingly have real-time access to their medical documentation.^22,23^

Given that increasing age comes with more opportunities to have heard these terms used in a medical context, it is somewhat surprising that older age was only associated with better understanding of 2 of the 13 phrases. In fact, the lack of consistent predictors of understanding by the demographics we studied (ie, age, gender, and education) highlights the importance of using clear communication with all patients.

We hypothesized that respondents would interpret plain-language descriptions of medical events more correctly than jargon-based descriptions. Indeed, in both cases for which we assessed comparisons the nonjargon phrase was significantly more widely understood. However, it is worth noting that while nothing by mouth was better understood than NPO by nearly 7-fold, 1 in 4 respondents also did not understand the phrase nothing by mouth. Given that in everyday language we do not talk about the act of eating or drinking as taking something by mouth, perhaps the clearest way to indicate that a patient should abstain from oral intake is to simply say, “You should not have anything to eat or drink.”

### Limitations

Our study has several limitations. Though participation was open to all adult fairgoers who volunteered, there is likely a naturally occurring bias in selecting individuals who would visit a university research building during their visit to a fair. Additionally, this research building had a mask mandate to mitigate the spread of SARS-CoV2 at the time of study, whereas many other areas of the fair did not. This requirement may have further selected a nonrepresentative sample. Although education demographics are not available for visitors to the 2021 Minnesota State Fair, in the state as a whole, 50% of those older than 25 years are reported to hold an associate degree or higher, which is higher than in many other states.^24^ In our sample, 77% reported that level of attainment, demonstrating a bias toward participants with more education. However, it is worth noting that only 2 of the survey questions showed increased understanding with increasing education. Furthermore, if education is associated with a better understanding of medical jargon, our results likely represent an overrepresentation of the actual comprehension at a societal level, which indicates these phrases may be even less understood in the population as a whole. Additionally, adding a control group of clinicians taking the survey would have been helpful to validate the agreement behind what clinicians intend when they use these phrases, though the only phrase we can hypothesize where this may be used differently depending on the context is the term “impressive” when describing radiography, which conceivably a clinician might use to describe how quickly something healed. Finally, though our survey questions were thoroughly assessed for bias, in some cases, the answer choices were multiple choice (eg, good news, bad news, or don’t know), allowing a survey respondent to guess and provide answers that may not reflect their true understanding.

## Conclusions

Medical jargon remains a common source of confusion for patients, and care should be taken to avoid using it with patients to prevent misunderstanding. Many commonly used jargon phrases are associated with poor understanding by the general public, and more people understood jargon-free versions of common medical phrases than expressions using jargon. No significant differences were found between an audio and a written version of the survey indicating that future studies of jargon understanding may support the less time-intensive written survey approach. Future studies should continue characterizing the understanding of jargon among the public and testing recommended alternatives to improve our communication with patients.

## Notes

Supplement. **eAppendix. **Survey Used in Study **eTable.** Associations Between Correct Responses of Jargon Terminology and Demographics in Cross Section of General Public
