# Supplementary material for: Accuracy in Patient Understanding of Common Medical Phrases
Source: JAMA Netw Open. 2022 Nov 30;5(11):e2242972. doi: 10.1001/jamanetworkopen.2022.42972 (PMC9713608; doi:10.1001/jamanetworkopen.2022.42972)
Supplement: Supplement. — eAppendix. Survey Used in Study eTable. Associations Between Correct Responses of Jargon Terminology and Demographics in Cross Section of General Public [file jamanetwopen-e2242972-s001.pdf]

Supplemental Online Content

Gotlieb R, Praska C, Hendrickson MA, et al. Accuracy in patient understanding of common medical phrases. *JAMA Netw Open*. 2022;5(11):e2242972. doi:10.1001/jamanetworkopen.2022.42972

eAppendix. Survey Used in Study

eTable. Associations between Correct Responses of Jargon Terminology and Demographics in Cross-Section of General Public

This supplemental material has been provided by the authors to give readers additional information about their work.

© 2022 Gotlieb R et al. *JAMA Network Open.*

eTable. Associations between Correct Responses of Jargon Terminology and Demographics in Cross-Section of General Public

#	Phrase	Age	Gender	Education
1	"Your cancer screening test came back and the **results are negative**."	0.64	0.13	0.15
2	"We are halfway through your chemotherapy treatment and your **tumor is progressing**"	0.76	0.25	0.29
3	"Your urine tests are back and there were **bugs in your urine**"	0.32	0.27	0.14
4	"You will need to be **NPO** at 8 am"	0.19	**0.008†**	0.13
5	"Your chest X-ray was **unremarkable**."	0.17	0.39	**0.034†***
6	"Your **nodes are positive**."	0.91	0.34	0.50
7	"The findings on the X-ray were quite **impressive**"	**0.002†**	0.06	0.15
8	"Your **blood culture was negative**"	**0.029†**	0.78	0.84
9	"Patient's neuro exam is **grossly intac**t."	0.09	0.15	0.73
10	"You are to have **nothing by mouth** after 4 pm"	**0.002†**	0.13	**0.015†***
11	"I am concerned the patient has an **occult infection**."	0.51	0.80	0.81
12	"Have you been **febrile?"**	0.47	**0.021†**	0.24
13	"Your blood tests showed me that you do not have an infection in your blood."	0.54	0.69	0.22

†P-values are from multiple logistic regression models. Table 4 in the manuscript shows the adjusted odds ratios for the statistically significant associations.

*For question 5, 69.7% (n=53) of respondents without a bachelor's degree answered correctly, compared to 80.9% (n=55) of those with a bachelor's degree as highest education, and 90.0% (n=63) with a graduate degree. The odds of a correct response were higher for a graduate degree compared to no bachelor's degree (aOR (95% CI) = 3.45 (1.35-8.87), **p=0.01**) but not for a bachelor's vs no bachelor's (aOR (95% CI) = 1.64 (0.74-3.64), p=0.23). For question 10, 61.8% (n=47) of those without a bachelor's degree answered correctly, compared to 79.4% (n=54) with a bachelor's degree, and 85.7% (n=60) with a graduate degree. The odds of a correct response were higher for both graduate degree compared to no bachelor's degree (aOR (95% CI) = 3.33 (1.39-7.99), **p=0.007** and for bachelor's compared to no bachelor's degree (aOR (95% CI) = 2.23 (1.00-4.95), **p=0.049**).

© 2022 Gotlieb R et al. *JAMA Network Open.*

eAppendix. Survey Used in Study

Survey Used in the Study: When Impressive Means Bad and Unremarkable Means Good: Laypeople's (Mis)Understanding of Common Medical Jargon

- For the following questions, imagine you are the patient and your doctor is sharing information with you about your health. You will be asked what you think the doctor is telling you.
- The survey will use both multiple choice questions and options for you to write out answers. Please write as neatly as possible.
- The goal is not to get the questions right, but rather to capture how you would interpret what the doctor is saying to you.
- The questions do not connect to each other, so answer each question as if you are a new patient.
- Please do not go back to change answers to earlier questions based on later ones.

☐ By checking this box I confirm that I am over 18 years old, am able to read, write, and understand English, do not have special training in the medical field, and have never worked in the medical field.

Demographics

Age _______

What is your gender?

Female
Male
Non-binary
Other

What is your highest level of education?
Some high school
High school diploma or GED
Associate's degree
Some college, no degree
Bachelor's degree

© 2022 Gotlieb R et al. *JAMA Network Open.*

	Graduate or professional degree<br>Other
1.	Imagine you are a patient who has just had a series of blood tests and your doctor says the following:<br><br>"Your cancer screening test came back and the results are negative."
	What do you believe your doctor is telling you?<br><br>A. The tests indicate you likely have cancer<br>**B. The test indicate you likely do not have cancer**<br>C. I don't know what the doctor means<br><br>If you answered A or B, how confident are you that you understand what the doctor is trying to say?<br><br>A. Not confident at all<br>B. Somewhat confident<br>C. Quite confident<br>D. Certain
2.	Imagine you are a patient receiving chemotherapy for cancer and your doctor says the following:<br><br>"We are halfway through your chemotherapy treatment and your tumor is progressing."
	What do you believe the doctor is telling you?<br><br>A. Your chemotherapy is working well<br>**B. Your chemotherapy is not working well**<br>C. I don't know what the doctor means<br><br>If you answered A, or B how confident are you that you understand what the doctor is trying to say?<br><br>A. Not confident at all<br>B. Somewhat confident<br>C. Quite confident<br>D. Certain

© 2022 Gotlieb R et al. *JAMA Network Open.*

<table>
<tr><td rowspan="2">3.</td><td>Imagine your doctor tells you the following:

“Your urine tests are back and there were bugs in your urine.”</td></tr>
<tr><td>Please write a short summary of what you believe your doctor is telling you. You can write “don’t know” if needed.

I believe the doctor is telling me that ___________________________

How confident are you that you understand what the doctor is trying to say?

A. Not confident at all
B. Somewhat confident
C. Quite confident
D. Certain</td></tr>
<tr><td rowspan="2">4.</td><td>Imagine your doctor tells you the following:

“You will need to be NPO at 8 am.”</td></tr>
<tr><td>Please write a short summary of what you believe your doctor is telling you. You can write “don’t know” if needed.

I believe the doctor is telling me that at 8 am I ___________________________

How confident are you that you understand what the doctor is trying to say?

A. Not confident at all
B. Somewhat confident
C. Quite confident
D. Certain</td></tr>
<tr><td rowspan="2">5.</td><td>Imagine your doctor tells you the following:

“Your chest X-ray was unremarkable.”</td></tr>
<tr><td>Do you believe your doctor is giving you:

A. Good news
B. Bad news</td></tr>
</table>

© 2022 Gotlieb R et al. *JAMA Network Open.*

	C. I don't know what the doctor means<br><br>How confident are you that you understand what the doctor is trying to say?<br><br>A. Not confident at all<br>B. Somewhat confident<br>C. Quite confident<br>D. Certain
6.	Imagine you are a patient who has just had surgery for cancer. After surgery your doctor gives you your test results and tells you the following:<br><br>"Your nodes are positive."
	What do you believe the doctor is telling you?<br><br>A. You are clear of cancer<br>B. **The cancer has spread**<br>C. The cancer has not spread<br>D. Don't know<br><br>If you answered A, B, or C how confident are you that you understand what the doctor is trying to say?<br><br>A. Not confident at all<br>B. Somewhat confident<br>C. Quite confident<br>D. Certain
7.	Imagine your doctor tells you the following:<br><br>"The findings on the X-ray were quite impressive."
	Do you believe your doctor is giving you:<br><br>A. Good news<br>**B. Bad news**<br>C. Don't know<br><br>How confident are you that you understand what the doctor is trying to say?

© 2022 Gotlieb R et al. *JAMA Network Open.*

	A. Not confident at all<br>B. Somewhat confident<br>C. Quite confident<br>D. Certain
8.	Imagine your doctor tells you the following:<br><br>“Your blood culture was negative.”
	Do you believe your doctor is giving you:<br><br>**A. Good news**<br>B. Bad news<br>C. I don’t know what the doctor means<br><br>How confident are you that you understand what the doctor is trying to say?<br><br>A. Not confident at all<br>B. Somewhat confident<br>C. Quite confident<br>D. Certain
9.	Imagine you are reading a written report from your doctor and you read the following:<br><br>“Patient’s neuro exam is grossly intact.”
	If you were to read this in a note from your doctor, would you take this to be:<br><br>**A. Good news**<br>B. Bad news<br>C. I don’t know what the doctor means<br><br>How confident are you that you understand what the doctor is trying to say?<br><br>A. Not confident at all<br>B. Somewhat confident<br>C. Quite confident<br>D. Certain
10.	Imagine your doctor tells you the following:

	"You are to have nothing by mouth after 4 pm."
	Please write a short summary of what you believe your doctor is telling you. You can write "don't know" if needed.<br><br>I believe the doctor is telling me that at 4 pm I ___________________________<br><br>How confident are you that you understand what the doctor is trying to say?<br><br>A. Not confident at all<br>B. Somewhat confident<br>C. Quite confident<br>D. Certain
11.	Imagine you are reading a report from your doctor and you read the following:<br><br>"I am concerned the patient has an occult infection."
	What do you believe the doctor means by an **occult infection**?<br><br>Please write a short summary of what you believe your doctor is telling you. You can write "don't know" if needed.<br><br>I believe occult infection means ___________________________<br><br>How confident are you that you understand what the doctor is trying to say?<br><br>A. Not confident at all<br>B. Somewhat confident<br>C. Quite confident<br>D. Certain
12.	Imagine your doctor asks you the following:<br><br>"Have you been febrile?"

© 2022 Gotlieb R et al. *JAMA Network Open.*

	What do you believe the doctor means by "febrile?"<br><br>I believe the doctor is asking me if I have ______________________<br><br>How confident are you that you understand what the doctor is trying to say?<br><br>A. Not confident at all<br>B. Somewhat confident<br>C. Quite confident<br>D. Certain
13.	Imagine your doctor tells you the following:<br><br>"Your blood tests showed me that you do not have an infection in your blood."
	Do you believe your doctor is giving you:<br><br>**A. Good news**<br>B. Bad news<br>C. Don't know<br><br>How confident are you that you understand what the doctor is trying to say?<br><br>A. Not confident at all<br>B. Somewhat confident<br>C. Quite confident<br>D. Certain

© 2022 Gotlieb R et al. *JAMA Network Open.*
